# Robots in travel clinics: building on tourism’s use of technology and robots for infection control during a pandemic

**DOI:** 10.1186/s40794-023-00197-7

**Published:** 2023-08-01

**Authors:** Irmgard L Bauer

**Affiliations:** College of Healthcare Sciences, Academy - Tropical Health and Medicine, James Cook University, Townsville, QLD 4811 Australia

**Keywords:** COVID-19, Travel medicine, Medical technology, Robotics, Artificial intelligence, Human-robot interaction, Automation

## Abstract

The arrival of COVID-19 impacted every aspect of life around the world. The virus, whose spread was facilitated overwhelmingly by people’s close contact at home and by travelling, devastated the tourism, hospitality, and transportation industry. Economic survival depended largely on demonstrating to authorities and potential travellers the strict adherence to infection control measures. Fortunately, long before the pandemic, the industry had already employed digital technology, artificial intelligence, and service robots, not to keep the world safe, but to either bridge staff shortages or save costs, reduce waiting times, streamline administration, complete unattractive, tedious, or physical tasks, or use technology as marketing gimmicks. With COVID-19, offering social distancing and touchless service was an easy step by extending quickly what was already there. The question arose: could travellers’ acceptance of technology and robots for infection control be useful in travel medicine? COVID-19 fostered the rapid and increased acceptance of touchless technology relating to all things travel. The public’s expectations regarding hygiene, health and safety, and risk of infection have changed and may stay with us long after the pandemic is ‘the new normal’, or a new one approaches. This insight, combined with the current experience with robots in health and medicine, is useful in exploring how robots could assist travel medicine practice. However, several aspects need to be considered in terms of type of robot, tasks required, and the public’s positive or negative attitudes towards robots to avoid known pitfalls. To meet the crucial infection control measures of social distancing and touch avoidance, the use of robots in travel medicine may not only be readily accepted but expected, and implications for management, practice, and research need to be considered.

## Introduction

The last 100 years have seen over ten pandemics or outbreaks [1], many either spread by travel or impacting travellers to outbreak destinations. Although tourism plays a major role in spreading infectious diseases, it is also in prime position to curb transmission by implementing a wide range of physical, structural, logistic, and technological means [2]. As early as September 2020, the World Travel and Tourism Council published ‘*To Recovery and Beyond’*, covering a comprehensive range of implications and recommendations [3].

COVID-19 was not just a medical emergency caused by a then little-known virus. It culminated in travel restrictions and bans across soon to be closed international borders and within countries. The virus threatened the survival of a multi-billion-dollar industry that exists solely by people moving about, congregating and meeting others at destinations around the world. An industry held hostage by the likes, dislikes, preferences, and attitudes of the travelling public must do everything to attract and keep customers.

Without a recent precedence, hence lacking theoretical guidance on the subject [4, 5], tourism started research immediately to participate actively in the control of the virus in the industry’s best interest. Much work aligned with the expertise of individual researchers and groups, and covered economic and managerial aspects but, importantly, a large section focused on people, the lifeblood of the industry, to develop appropriate strategies. These strategies are of interest to travel health professionals as they address travellers’ health and demonstrate how another industry responds to health directives. Research covered a wide variety of topics: risk perceptions and travel intentions, mass gatherings, physical and psychological impacts on tourism employees and residents at destination, hostility and discrimination, as well as the use of technology to meet health directives and so mitigate tourism’s role in spreading the virus [4].

Fear of infection was reinforced when, at the beginning of the pandemic and with much disquietude, pictures went around the globe of people in masks and face-shields, puzzling use of gloves, horrific scenes in intensive care units, or people in full PPE spraying vigorously bushes and cars in urban streets. Suddenly, it became critical to avoid touching surfaces and people, being coughed and sneezed on and, preferably, to stay away from others altogether. Technology, used widely before, became the supreme means to assist humans in daily life.

Artificial intelligence-driven services have been everywhere in recent years in tourism and transportation, including travellers’ source of information from government websites, news outlets, local information to alert to natural, political, or other potential threats to safety and security, and health advice. Complete travel and travel health advice can be uniquely tailored to traveller and trip (with caution) by ChatGPT. Travel medicine already uses smartphones for research, risk assessment, medication monitoring, and consultations. Like in infectious disease control, phones can be used for communicating symptoms, advice or pictures to assist diagnoses [6]. These uses are quick and convenient, if impersonal. However, there are still many situations where some resemblance of face-to-face contact mirrors the notion of service. This is where robots can step in – and add to infection control.

This perspective first presents tourism and hospitality’s use of technology, including robots, to meet the demands of authorities combatting the spread of COVID-19. This is important for travel health professionals who release ‘their’ travellers into the world where contactless activity is an extension of their advice regarding infection avoidance. Furthermore, assuming that COVID-19 is not the last pandemic to bring the world to a standstill, safe robot use during travel should be part of pre-travel care. Collective experiences and insights of robot use in tourism (and healthcare) encourage robot employment in travel clinics as a logical and seamless step towards modern infection control.

## Method

The search for literature, predominantly post-2020, utilised PubMed, Scopus, Web of Science, ScienceDirect, Google Scholar as well as grey literature with combinations of search terms: ‘robot’, ‘automation’, ‘human-robot interaction’, ‘tourism’, ‘travel’, ‘health’, ‘medicine’, ‘COVID-19’, ‘infection control’ and included only English-language material. Reference lists of attained papers provided further sources.

## Technology in tourism and hospitality: its new role in infection control

Technology has long played an important role in front-of-house tourism, starting possibly with the replacement of paper plane-tickets with eTickets. Since then, rapidly evolving technological advances found their practical use in tourism and hospitality: cashless payment, self-serve kiosks, self-check-in at airports or hotels, digital IDs, passports and vaccination certificates, drones delivering food, autonomous vehicles, service robots, and smartphone applications for destinations, as tour guides or for crisis communication. Convenience and speed for customers stood opposite cost saving and staff reduction for companies. The arrival of COVID-19, and the fear of infection, accelerated the use of technology as there was now a multitude of ways to avoid touch and keep distance.

The perfect infection control, of course, is: not going away. Virtual travel (VT) existed through television travel programs and became more sophisticated over time with webcam-travel, virtual tours and, more recently, vTime, an immersive virtual reality world controlled via one’s head movements. Fixed cameras at popular destinations transmit live pictures; curated ‘tours’ through museums and exhibitions, nature, attractions, towns, and activities allow visits without being there. VT, free or purchased, is inexpensive and safe, and benefits people who are unable to travel, e.g., the disabled or infirm. It can accommodate those who need to follow socio-cultural and religious rules and meet gender-based role expectations [7]. It is not ‘real’ travel but may tempt viewers to visit a destination in the future [8]. VT can enhance mental well-being, especially during lockdowns in a pandemic [9, 10], and for those with perceived high threat severity [11]. Presumably, it may do the opposite and highlight cultural restrictions for those who cannot travel freely. During COVID-19, VT was a little something for would-be travellers who had nowhere to go, or people confined to their homes during lockdown. It was excellent for infection control but no salvation for a panicked industry, nor did local communities benefit [4].

Food and drink evoke a particularly high expectation regarding hygiene standards. Robots have cooked meals for some time [12]. In 2014, Royal Caribbean International opened the Bionic Bar with twin robots preparing drinks on request, appropriately programmed after the aesthetic movement of a principal ballet dancer [13]. Seen at the time as ‘cool’ gimmicks, the pandemic encouraged the use of technology to meet the requirements for social distancing and a touchless existence. Self-serving food technology has a long history creating a convenient 24/7-availability of goods without paying for staff. Now, self-service food kiosks meet diners’ expectations not only because of shyness, perceived control, or intolerance for tardy service, but the perceived reduced risk of infection from an employee [14]. The need to touch a touchscreen can be overcome (pers. obs. IB) by keying instructions inventively with knuckles or elbows, through a layer of tissue or a stylus pen. Food-delivery apps arrange delivery by people at a distance [15] or to a building or floor storage unit and from there by robot to the customer apartment [12]. Drone food-delivery works similarly [16]. If patrons venture out to eat, robots greet, take orders, and deliver meals [12].

Among all tools available, robots stand out for their sophistication (compared to self-services) and their potential to replace certain human actions. After a brief section on robots and a summarised presentation of their employment in tourism, hospitality and health, this perspective discusses how robots may be of use in travel clinics.

## Robots

Robots are machines that perform automatic tasks guided by different levels of autonomy. They are to serve us (Old Slavonian *robota* = servitude) by completing tasks that are dull, dirty, or dangerous. They still lack emotion and social skills but provide consistent precise action. Robots require sensors (vision, touch, sound, smell, taste), actuators (whole-body motion, manipulation) and computational capabilities [17]. Robots ‘work’ in innumerable roles, such as in manufacturing, medicine and health care, the military, including mine clearing, coral husbandry, autonomous underwater locomotion, or research on Mars.

The idea of automated devices to complete physical tasks goes back to antiquity. To the delight of onlookers, dead objects like dolls, figurines or machines became alive and danced, moved head and limbs, played instruments, or served tea. Leonardo da Vinci’s mechanical knight, Chinese clocktowers and many other human-like automata come to mind. Not quite a *perpetuum mobile*, an automaton was driven by water or steam. Later, air pressure powered robots in movies, e.g., Fritz Lang’s *Metropolis*. The arrival of electricity created industrial robots, predominantly in manufacturing, that worked with consistent precision without needing a break. Since then, the field of robots, robotics and their applications has grown exponentially thanks to fuzzy logic and artificial intelligence which drive all modern robotic devices.

### Fuzzy logic and artificial intelligence (AI)

Fuzzy logic, in contrast to classic logic which uses statements of absolute truth, processes sets of relative truths and levels of possibility, using algorithms to decide similar to human problem solving. Conditions are a matter of degree rather than precision. First mentioned by Lotfi Zadeh in 1965 [18], robots use fuzzy logic to sense information from their environment and respond based on a decision-tree analysis, allowing a much wider scope of applicability. Fuzzy logic is at the core of the rapidly evolving field of AI. According to one of the co-founders of the discipline, AI is ‘the science and engineering of making intelligent machines, especially intelligent computer programs’ [19, p.2]. Such programs are capable, like humans, to communicate, store information, use information to draw conclusions, extrapolate patterns, perceive objects, and manipulate objects and move about [20]. AI drives anything from search engines, speech recognition, automated cars to composing essays or answering questions. AI fuels robots, and the better the program, the more sophisticated the robot. Despite the enormous range of useful applications, there are concerns about potential misuse and loss of human control. Over ten years ago, while all important parts were there, there was ‘still something important missing in the overall picture’ [17, p.294]. Today, AI makes robots appear to ‘think’. At the time of writing, ChatGPT, a large language processing tool which produces AI-created texts based on knowledge available up to 2021, appeared in the media around the world highlighting its benefits and risks. However, uncritical reliance on the tool is fraught with problems. For example, to the prompt ‘in a sentence, describe the weaknesses of ChatGPT’ it responded ‘ChatGPT’s weaknesses include a lack of common sense, context-specific knowledge and a tendency to generate nonsensical or biased responses’ [21]. Theoretically, ChatGPT-text could be voiced by a robot.

There are multiple applications of clinical AI, but patients’ and the public’s perceptions and attitudes vary greatly. AI is viewed positively with some reservation for diagnostic purposes but should not replace clinicians or their supervision. AI can serve as a second opinion and complement a physician but, in the case of conflict, trust rests with the clinician [22]. Trust is also greater if the system is set up by health care rather than technology companies. Clinical AI has strengths but also weaknesses: physicians may not approve of patients ‘supplementing’ their care with AI, and potential legal and ethical consequences [22]. AI should help clinicians but not decide, act or recommend [23]. Members of the public placed equal trust in a diagnosis by physicians and AI, but trusted AI more for cancer diagnoses and would be willing to pay for an AI-review of medical imaging. There was no taste for unsupervised autonomous robotic surgery [24]. AI in tourism and hospitality is equally widespread, found anywhere outside the traditional face-to-face contact and in all examples described later in this article.

### Humanoid robots

From human-like automata and robotic arms assembling cars, it was only a small step to create robots with human appearance and locomotion. From a box with a head to a torso with head and arms, to a full-sized humanoid capable of relatively smooth movements, the aim was to create anthropomorphism to the point that robots looked uncannily like humans. But there was a stumbling block. The ‘uncanny valley’, first explained in 1970 by Masahiro Mori [25], describes how a person’s positive response to obviously artificial gadgets suddenly changes to repulsion when a realistic looking robot that smiles unnaturally and whose eyes may even follow a person, just looks creepy. This is similar, for example, to a myoelectric hand which looks like an amputated hand that still moves. Amazing from an engineering point, employing uncanny humanoid robots requires careful consideration. People were more accepting of a humanoid robot when told that it was controlled remotely by a person, rather than the robot acting autonomously [26]. Moral decisions of robots that appear eerily human were judged less moral compared to the same decisions by non-humanoid robots or humans [27]. There may be a greater acceptance by ‘gadget-nerds’ or younger people, but due to the negative response to the uncanny valley effect, human-like robots should be employed with care where people’s positive response is crucial, as in health and medicine, and tourism and hospitality. As a world-first, in 2017, Saudi Arabia granted citizenship to the English-speaking, non-Muslima human-like SOPHIA [28].

### Robots’ impact on job security

The World Economic Forum predicted that by 2025, 85 million jobs will be replaced by technology, but 97 million new roles created with humans, machines and algorithms working together [29]. While this trend will have benefits, many such roles will not suit low-tech local jobs, and opportunities for local tourism workers will be lost [20]. During COVID-19, robots filled in for missing employees but in turn, created redundancies. Robots induced unemployment [30]. Travellers who liked robots for service provision did not worry about possible social costs and job losses [31].

## Robots in tourism and hospitality – tools for infection control

Service robots are connected to a company-wide system and interact, communicate, and deliver services to this company’s customers [32]. Drivers to adopt automation are technological progress, labour shortages (also due to pandemics), customer demand and expectation, and innovative capabilities [33]. Rapport, trust, and usage intention, i.e., human-robot interaction, depend on robots’ perceived intelligence, social presence, and social interactivity [34]. AI-driven systems and robots, e.g., the world’s first social robot PEPPER, or concierge CONNIE and many others, have long been employed in tourism and hospitality. Examples are too numerous to list but include travel information, booking, airline self-check-in, bag drop, automated border control, customer service, hotel check-in and check-out, welcoming guests and taking, storing and delivering luggage, room service, vacuuming, cleaning, security checks, entertainment, concierge services, and generally reducing waiting times [35–37].

A recent study of Egyptian domestic tourists suggested that positive visitor satisfaction, based on emotional well-being and perceived safety, and health consciousness both led to a willingness to use service robot [38]. Before the pandemic, robots received a mixed report card because many viewed them as non-anthropocentric, not what travel is about [39]. With COVID-19, their importance rose immediately [40]. Robots, robotic vehicles and other autonomous devices have been used in airports, recreation areas, and hotels and restaurants [36]. Much research into their accelerated use and acceptance by travellers hoped to demonstrate that robots project a lower risk of infection [41] and people would again travel more [42]. Robots’ ability to be cleaned and sanitised frequently certainly helped. However, robotic service was seen positively only in economy, not full-service hotels [43]. They were more acceptable in utilitarian services, e.g., transport, and not so much in hedonic services, e.g., hospitality supposed to provide pleasure and enjoyment [44]. People’s views on facemasks translated to anthropomorphic robots with and without facemasks, representing a shared subjective experience [45]. One was also safe from moral judgment from robots to embarrassing requests; however, this changed with increasing anthropomorphism [32]. Pre-COVID-19, uncannily humanlike robots in hotels triggered uncomfortable feelings (‘creepy’, ‘weird’) and concerns about perceived safety [46], especially when mortality is salient [47]. In contrast, Gen Z thought the more humanlike a robot, the better the infection control [48].

29,507 TripAdvisor reviews of 80 hotels worldwide using robots for various tasks yielded mixed acceptance, especially due to disappointing performance, malfunction, or ‘creepy’ appearance [35]. In the US and UK, cleaning robots were seen as less competent than human cleaners unless the task was disgusting, e.g., urine on the hotel floor or blocked toilets [49]. Apart from data and privacy concerns, robot failure is a major setback, most spectacularly demonstrated in the Henn na Hotel where, among many malfunctions, robots mistook guests’ snoring as cries for help and acted as programmed [50].

Pre-pandemic, a study suggested customers are more critical of human service failure than robot failure as human performance is prone to inconsistencies [51]. Cuteness of robots raises failure tolerance, but humour expressed by a robot is only acceptable in low-severity mishaps; the more anthropomorphic a robot, the more negative the perception of its humour [52]. Robots may mitigate discrimination by and of employers, employees, and tourists by serving without judgments but may indirectly discriminate by robots representing a particular race or gender or against people who are unfamiliar with the use of robots and need to ask for help [53, 54]. Post-COVID-19, a combination of human, robotic and mixed service in tourism and hospitality was envisaged [55], but ‘contactless service’ still must focus on ‘service’, not just ‘contactless’ [56]; a move from high-touch-low-tech to high-tech-low-touch may be detrimental to an industry that is built on human interaction [37].

## Robots in health care and medicine

Health professionals are familiar with the use of robots. Automated patient-support has existed before, especially in health kiosks, publicly accessible computing devices, providing a range of services including health information, clinical screening, self-check-ins, telehealth, or medication monitoring in general as well as specialty and outpatient clinics. Despite the arrival of robots, health kiosks will still be needed for a long time [57].

In healthcare, robots assist clinicians, direct users, and caregivers with tasks inside, on and outside the body [58]. Micro-robotics dispense or remove material in the body or act as sensors. Robots assist in ever advancing surgical procedures and sonography, or as patient simulators. Robotic prostheses, orthoses, and exoskeletons replace missing limbs, while other robots assist with physical tasks, also in contaminated environments [58]. Robots assist nurses, caregivers, and individuals in domestic and medical tasks, including lifting/transferring patients, assistance with personal care, medication management, meal delivery, vital sign measurement, call for help, household tasks, cleaning, disinfecting and waste disposal, escorting, companionship, or dog walking [59]. Robots transport patients and goods, and complete hospital admissions or discharge. Robots have been successful in reducing children’s dental anxiety [60], pain and distress regarding vaccinations [61], including parents’ anxiety [62], and in self-management education for children with Type 1 Diabetes [63]. PEPPER seemed helpful in combatting influenza vaccination hesitancy through health education, though this study did not include a control group [64]. The robotic baby harp seal PARO has been used in many aged care facilities to improve biopsychological conditions, especially in dementia patients. The autonomous robot, programmed with five senses, has been partly useful [65], but should not replace staff time [66]. No doubt, many more applications are in development, such as hair-washing robots [67], a venepuncture robot to address rolling veins [68] or a smart robotic crutch [69].

While the technical abilities and applications are one aspect, the acceptance and perceived usefulness by users and staff are another, regardless of solutions to manpower concerns [70]. In one study, users’ views varied depending on the task but were similar across all age-groups (18–98 y) [59]. Staff found social robots beneficial and practical in psychosocial care for older adults in long-term facilities [71]. A systematic review of human and robot personality in health care suggested that a matching personality of both was a key predictor of whether patients accepted a robot as health care worker. A robot’s personality included extroversion, femininity, playfulness, or seriousness [72].

## Robots safeguarding travellers’ health

COVID-19 not only devastated the tourism industry; there were few to no travellers needing travel health care. People’s fear of infection through close contact with others or touching contaminated surfaces, and their expectations that their well-being was safeguarded, changed dramatically with this pandemic. Travel medicine discussed present and future aspects of COVID-19 [73, 74], but there is little evidence of looking over the fence to see what others, especially tourism, do and how to benefit from such strategies.

The appreciation of robots for travellers’ health is important for two reasons. First, clinicians should be familiar with how robots control infection in tourism settings, if they are employed correctly or as mere tokens, if any content they convey is correct, and how touchscreens are disinfected. The latter has been a point of contention for as long as touchscreens in computers, phones, food kiosks or in-seat entertainment on planes have spread pathogens from one user to the next. Fomite-based transmission of microbes, e.g., *Staphylococcus aureus, Escherichia coli* or *Clostridum difficile*, has been studied at large [75, 76]. It is impractical to clean a screen after each user. Therefore, one should remind travellers to wash or sanitise hands after touching a public screen, typically an automatic action without further thought. Travellers may also ask about service robots’ benefit or trustworthiness in infection control or request practical tips. Such conversations lead to research questions, preferably for multidisciplinary teams: How do travellers perceive the value of robots during a pandemic? Are individuals’ levels of anxiety regarding COVID-19 associated with attitudes towards robots? Does the use of robots change patient-clinician relationships? How does technology change travellers’ risk perception? What are views and acceptance of robots by clinic staff? In addition, travel medicine should collaborate with tourism regarding the correct purpose, actions, program content, and hygienic maintenance of service robots.

Second, the rapidly evolving acceptance of technology may make the use of robots in clinics not only useful but provide a continuum in control measures from pre-travel care to travel. Travellers are in the clinic for minutes, in the sphere of tourism for days, weeks or months.

## Robots in travel clinics

Current insight into robots and research into their use in tourism and in health allow clinicians to explore possible uses of suitable robots in clinics and surgeries. Experience with service robots elsewhere provides a basis for considering the possibility of employing a robot while taking advantage of the benefits and avoiding pitfalls, especially around privacy and patient data protection. Based on the literature presented earlier, some suggestions follow.

### Decision to employ a robot

People expect visible evidence that a health facility adheres to hygiene requirements, especially in a pandemic. The presence of a robot may further reinforce public health directives on distancing and touch avoidance, and influence travellers’ behaviour long after they left the clinic. Robots also protect staff health.

### Staffing issues

Robots are often used to replace staff and save costs. This is not a robot’s purpose in travel medicine. With the widening of the specialty, travel medicine literature suggests ever more content to be included in travel health care in often severely limited timeslots. The appropriate use of robots is enormously timesaving, freeing clinicians, and especially nurses, to devote their limited time to quality care tailored to the traveller. A robot is employed in addition to staff, not instead of staff.

### What is the robot supposed to do?

Robots can solve problems based on their algorithm and solution sets. They can only do what they are programmed for. Designing the appropriate AI is a task for programmers and engineers; travel health practitioners provide the correct input. A robot is particularly useful in pre-travel care where it deals with time-consuming pre-consultation questionnaires, post-consultation outcome-‘tests’, travel health advice, and general health education. As elsewhere, it could be used to address children’s (and parents’) anxiety regarding travel vaccinations. A robot can also clean and disinfect, or transport vaccines, medication [77], or travel gadgets a clinic may sell. Tasks completed by robots elsewhere could be copied where appropriate. A robot can greet patients and, on exiting a clinic, wish: ‘safe travels!’. During travel, depending on a clinic’s robot use, data could be transferred to or from a mobile platform [60] and serve in any of the functions where smartphones are currently used.

### Choice of robot

Once the required tasks have been decided, the choice of robot must be made, especially if it should be non-hominoid, or hominoid, with caution against human-like appearances. It can be simple, e.g., like PEPPER with a touchpad and screen, or front panel allowing for animation or playback, or it could be very sophisticated, depending on the requirements and its degree of autonomy. The output can be text or voice. Robots can be embellished as a clinic feels appealing or appropriate, without it being turned into a cheap gimmick.

### Marketing

The careful choice of robots and their role may prove an excellent marketing tool if travellers see the robot as value-enhancer. This applies not only to ‘tech-nerds’. Children may insist on going to ‘the clinic with the robot’, a definite benefit, when it comes to essential repeat visits for vaccinations.

### Maintenance and cost

Most importantly, the robot must work; failures are not acceptable. Regular maintenance and program updates need to be factored in, as well as a suitable disinfection method for robot and touchscreen [78]. Cost is an issue in all medical setting, especially in smaller clinics. However, the cost of a robot is still much less than the annual cost of a staff member. Alternatively, robots can be leased. Staff training needs to be costed as well.

## A Robot for the Road?

Finally, what about robots for travellers? The pocket-sized ‘Cleansebot’ promises to be an ‘automatic germ-killing machine for your travels’. It kills ‘germs and bacteria’, making a room ‘a little bit cleaner’. It slips between bedsheets to attack any unpleasantness left by a previous guest and combats airborne viruses, presumably by waving the gadget in the air. The makers, unburdened by scientific insight into microbes, refer to test results [79] which are unable to be located. There is currently no robot for use ‘on the road’.

## Conclusion

The appearance of COVID-19 at the beginning of 2020 was not only a medical emergency but a question of survival for the tourism industry. Like health care, tourism and hospitality are ‘high-touch’ industries where close contact with people is at the core of service.

With distancing and touch avoidance, the main infection control measures utilised technology, automation, and robots to ensure that service was delivered as best as possible under the circumstances, even though it is methodologically difficult to provide evidence for case reduction. For a long time, robots have proved useful in many fields for a wide variety of purposes. During the pandemic, their role as a ‘go-between’ in transactions between service provider and recipient reached a new significance.

The current paper utilised the first studies on technology during COVID-19. There will be many more on robots as infection control in tourism and in health. Based on current (and future) evidence, travel medicine may benefit from becoming part of a network of service robot providers for the benefit of travellers and practitioners. A more automated future may provide a seamless link between pre-travel health care and travellers’ experiences with technology during their trips. A new pandemic might just be around corner.

## Data Availability

Not applicable.
